# Systems and Photosystems: Cellular Limits of Autotrophic Productivity in Cyanobacteria

**DOI:** 10.3389/fbioe.2015.00001

**Published:** 2015-01-20

**Authors:** Robert L. Burnap

**Affiliations:** ^1^Department of Microbiology and Molecular Genetics, Oklahoma State University, Stillwater, OK, USA

**Keywords:** cyanobacteria, growth rate, molecular crowding, optimization, photosynthesis, ribosomes

## Abstract

Recent advances in the modeling of microbial growth and metabolism have shown that growth rate critically depends upon the optimal allocation of finite proteomic resources among different cellular functions and that modeling growth rates becomes more realistic with the explicit accounting for the costs of macromolecular synthesis, most importantly, protein expression. The “proteomic constraint” is considered together with its application to understanding photosynthetic microbial growth. The central hypothesis is that physical limits of cellular space (and corresponding solvation capacity) in conjunction with cell surface-to-volume ratios represent the underlying constraints on the maximal rate of autotrophic microbial growth. The limitation of cellular space thus constrains the size the total complement of macromolecules, dissolved ions, and metabolites. To a first approximation, the upper limit in the cellular amount of the total proteome is bounded this space limit. This predicts that adaptation to osmotic stress will result in lower maximal growth rates due to decreased cellular concentrations of core metabolic proteins necessary for cell growth owing the accumulation of compatible osmolytes, as surmised previously. The finite capacity of membrane and cytoplasmic space also leads to the hypothesis that the species-specific differences in maximal growth rates likely reflect differences in the allocation of space to niche-specific proteins with the corresponding diminution of space devoted to other functions including proteins of core autotrophic metabolism, which drive cell reproduction. An optimization model for autotrophic microbial growth, the autotrophic replicator model, was developed based upon previous work investigating heterotrophic growth. The present model describes autotrophic growth in terms of the allocation protein resources among core functional groups including the photosynthetic electron transport chain, light-harvesting antennae, and the ribosome groups.

## How Fast Can Cyanobacteria Grow?

**Figure 1:**
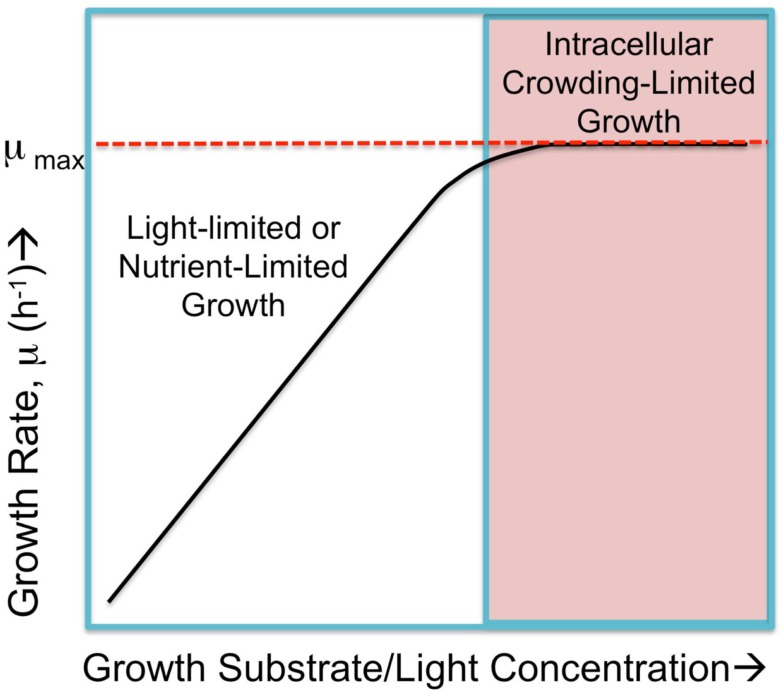
**Hypothetical cyanobacterial growth rate, μ, in response to substrate abundance falls into two regimes**. Submaximal rates of growth occur when either the light intensity or nutrient availability is limited and growth rates increase in proportion to increases in the limiting commodity, as shown in the left portion of the hypothetical graph. When these are not limiting, the growth rate is the saturated, maximal rate, μ_max_. This maximal rate is hypothetically limited by physical constraints such as packing all necessary molecular machinery, small molecules, and ions into the confined space (cytoplasmic and membrane) of the cell yet have small enough cell dimensions to allow sufficient nutrient exchange. Cells approach a maximal rate where internal factors, referred to as “intracellular crowding-limited” dominate. Figure adapted from O’Brien et al. (2013), but the “macromolecular expression (ME)-limited” or “proteome-limited” growth rate is here considered as limited intracellular crowding as discussed in the text.

There is a very wide range of maximal growth rates observed among cyanobacterial strains (Carr and Whitton, 1982). Fast growing model cyanobacterial strains can be grown with doubling times in the range of 3–6 h under optimal conditions (Binder and Chisholm, 1990; Nomura et al., 2006; Kim et al., 2011; Ludwig and Bryant, 2011). On the other hand, many cyanobacterial strains have doubling times on the order of once per day (Carr and Whitton, 1982). Moreover, even the fastest growing cyanobacteria are still much slower growing than many heterotrophic bacteria and yeasts. Furthermore, the factors accounting for the diversity of maximal rates of cyanobacterial growth remain poorly understood and it appears that autotrophic growth tends to be slower than heterotrophic growth, which can be as short as ~10 min doubling times (Labbe and Huang, 1995). This is important because researchers often include “fast growth” among the criteria in choosing a cyanobacterial strain for engineering. Since the fastest rate of growth in heterotrophs occurs in “rich” media containing abundant amino acids and cofactors and since the main macromolecular investment in cell growth is the synthesis of proteins, then a reasonable hypothesis is that autotrophic metabolism in cyanobacteria results in slower maximal growth because of the necessity for the synthesis of the amino acids and all other cell components from CO_2_. However, assuming it is the burden of synthesizing amino acids, then the question arises whether this is because of the energetic cost of making amino acids, such as ATP consumed per amino acid and “opportunity costs” of not using ATP for other cell functions that contribute to cell reproduction. A bioinformatics analysis using codon bias as an indicator for expression rates found that less expensive amino acids are preferably utilized for highly expressed protein (Akashi and Gojobori, 2002). However, energetically more expensive amino acids also tend to require more biosynthetic steps, and consequently a greater number of enzymes. Moreover, a more exhaustive analysis found that amino acid utilization rates for protein were only weakly correlated, if at all, with the bioenergetic costs of their synthesis (Barton et al., 2010). Or is it something else, such as greater cellular space devoted to the corresponding biosynthetic enzymes? As discussed below, recent theoretical and experimental studies point to the latter and suggest that the ultimate speed limit relates to the physical constraints of packing all necessary molecular machinery, small molecules, and ions into the confined space (cytoplasmic and membrane) of the cell yet have small enough cell dimensions to allow sufficient nutrient exchange (Figure 1). Analysis of the physical state of cytoplasmic water in *E. coli* under different osmotic conditions indicates that macromolecular crowding limits growth rate, probably through decreasing the “kinetics of some biopolymer diffusion processes” (Cayley and Record, 2003). Accordingly, intracellular crowding appears to be the main constraint to growth and sets the upper bound on the size of the proteome. Evidence for this includes the analysis of the impact of protein overexpression on maximal growth rates (Scott et al., 2010; Scott and Hwa, 2011; You et al., 2013) and the effects of crowding on diffusion within the bacterial cell (Klumpp et al., 2013; Soh et al., 2013; Parry et al., 2014). Crowding also would affect the size to the metabolome, primarily because of the limited amount of free water, as discussed below. Based on these considerations, the total size of the proteome is likely bounded by intracellular crowding constraints and, assuming a relatively fixed amount of dissolved ions and metabolites^1^, the allocation of proteomic resources becomes a “zero sum game.” This is one of the key points for this discussion since we are trying to understand the physical basis optimal proteomic allocation strategies. As discussed below, the results of modeling studies and the consideration of “overflow” metabolism is best explained by a limitation in the total amount of protein that can be crowed into a cell, yet remain soluble and diffusionally mobile. From that perspective, it could be that the autotrophic lifestyle requires a comparatively large investment into photosynthetic and other anabolic proteins. This would include the large investment into extensive internal membranes for photosynthetic machinery as well as the need for large amounts of the catalytically inefficient carbon-fixing enzyme, Rubisco. Correspondingly, this comparatively greater investment would come at the expense of the macromolecular machinery dedicated to cell duplication including ribosomes, initiation factors, cell division proteins, and all the metabolic precursors required for the duplication. Conversely, it might be expected that very fast growing bacteria (Labbe and Huang, 1995) have a greater investment in the machinery for cell duplication and a streamlined metabolic capacity that constituted by a minimal set of core enzymes, transporters, and metabolites for the provision of precursors to support the operation of the cell duplication machinery.

## Engineering and Modeling Cyanobacteria

Because of their comparative cellular simplicity and ease of genetic manipulation, cyanobacteria are the object of numerous biotechnological efforts for metabolic engineering for the renewable production of biofuels and high-value products. Compared with algae and plants, cyanobacteria are easier to genetically modify and are amenable to organism-wide metabolic modeling, which are attributes that lend themselves to synthetic biology approaches [for current review see Berla et al. (2013)]. Cyanobacteria have thus become the targets of biofuel production that includes the engineered production of ethanol (Deng and Coleman, 1999), butanol (Lan and Liao, 2011), isoprene (Lindberg et al., 2010), ethylene (Ungerer et al., 2012), sugars (Ducat et al., 2012), and lactate (Angermayr et al., 2012). Besides biofuel production chemical feedstock, high-value compounds, and “nutraceutical” products are envisioned and important progress has been made (Xue et al., 2014).

In parallel with this progress in genetic engineering of desired physiological characteristics into cyanobacteria, systematic modeling approaches for understanding the productivity of genetically modified cyanobacteria have been developed. To gain insight on the physiological characteristics of the strains targeted for engineering studies, researchers have developed large-scale models of metabolic networks based upon annotated gene content that has been deduced from genomic sequences for a number of both heterotrophic and autotrophic bacteria [technical approaches reviewed in Covert et al. (2001), Feist et al. (2009), Steuer et al. (2012)]. Such models can include hundreds and thousands of enzyme-catalyzed reactions based upon the predicted gene content of an organism. The term “metabolic network re-construction” is used to describe the process of developing such models. The successful application of this approach is time-consuming and requires, among other things, careful manual evaluation of algorithmically assigned gene annotations and strategies for “filling in” predicted enzymes when the corresponding genes are missing from the genomic analysis. However, the rewards for developing a robust metabolic re-construction appear to be large (Knoop et al., 2010, 2013; Nogales et al., 2012). When combined with linear programing methods, the network models can be used for predicting, *in silico*, the metabolic flux patterns under different assumed environmental conditions using linear programing methods termed flux balance analysis (FBA). Of the possible computational approaches, constraint-based FBA has already proven useful and appropriate for the predictive analysis of the production of engineered bioproducts (Nogales et al., 2013). FBA has been used for the theoretical evaluation of photosynthesis in cyanobacteria under different trophic conditions including photoautotrophic, photoheterotrophic, and heterotrophic growth (Knoop et al., 2010, 2013; Nogales et al., 2012). Recently, the theoretical yields of different excreted products such as butanol and sucrose from engineered cyanobacteria have been analyzed and the computational models give predicted values that are close to experimentally observed yields (Nogales et al., 2013). One of the conclusions for that analysis is that autotrophic metabolism in cyanobacteria is relatively inflexible with respect to genetic engineering and that the network properties of autotrophic metabolism place basic limitations on the yields of engineered products.

There are also important experimental applications for reconstructed metabolic networks. This includes metabolic flux analysis (MFA), which uses pulse-labeling of cells with substrate atoms tagged using stable isotopes. This approach has been applied in cyanobacteria to analyze metabolic fluxes under autotrophic conditions (Young et al., 2011). Again, metabolic network models are used, but they are used as input to fit the experimental labeling patterns (Young, 2014). Thus, metabolic network re-construction is of utility from both the experimental and theoretical perspectives and allows insight into the details and global features of microbial growth and metabolism.

## Course-Grained Models of Microbial Metabolism

Besides the detailed and metabolically realistic FBA models mentioned above, simpler “course-grained” modeling approaches to understanding growth and global features of microbial growth have been developed. Of these, the models of heterotrophic microbial growth (Molenaar et al., 2009; Scott et al., 2010; Scott and Hwa, 2011) have lent themselves to comparison to the corresponding FBA models. In some respects, the simpler alternatives for heterotrophic microbial growth and metabolism have produced explanatory results that were not sufficiently addressed the early FBA models^2^. Molenaar et al. observed, for example, which so-called “overflow metabolism” of microbial growth that was not adequately accounted for in the earlier implementations of FBA (Molenaar et al., 2009). Overflow metabolism is a form of metabolic energy spilling involving the excretion of energy-rich precursors by heterotrophic microbes under certain trophic conditions (discussed below). The course-grained models of Molenaar et al. explicitly take into account the limitation of a finite proteome and frame the problem in terms of a self-replicator cell growth model that optimizes the allocation of finite proteomic resources to maximize growth rates (Molenaar et al., 2009). These models optimize the allocation of cellular protein resources in a small set of general cellular functions such as transport of substrate into the cell, conversion of substrate into metabolic precursors for protein and lipid synthesis, and though overly simplistic, they were able to reach some key conclusions that are consonant with more sophisticated FBA approaches (O’Brien et al., 2013). Both the course grained and FBA modeling are most realistic when maximal growth rates are constrained by “proteomics costs” and the inclusion of this constraint allows for an accounting for certain counter-intuitive physiologic behaviors such as overflow metabolism.

**Figure 2:**
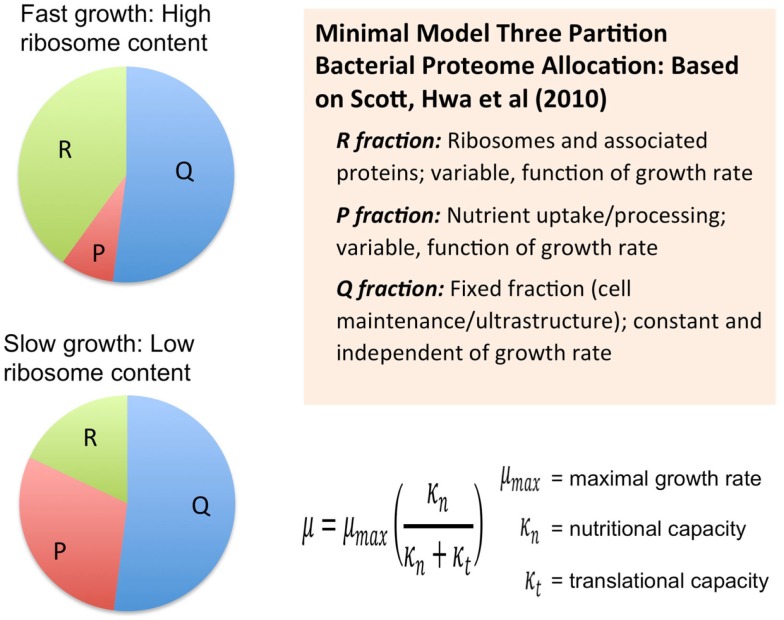
**Allocation of the proteome among different sectors as defined by the analysis of growth in the development of a phenomenological theory regarding its control (Scott et al., 2010; Scott and Hwa, 2011)**. Bacterial proteome consists of a fixed fraction, Q, whose proportion is constant and largely unaffected by the growth rate of the cells and may contain proteins for cell maintenance and ultrastructure. The remainder of the proteome minimally is partitioned into two additional fractions, R and P, which represent ribosome affiliated proteins and nutrient uptake and processing proteins, respectively. The R and the P fractions are observed to reciprocally change their proportions as a function of cell growth rate with the R fraction reaching its largest magnitude under fast growth conditions. This coincides with the observation that ribosomes are more abundant in faster growing cells compared to more slowly growing cells. This same theory was used to derive a Monod-like relationship of observed growth rate in relationship to nutrient availability as reflected by parameter κ_n_, along with the translational capacity represented by the parameter κ_t_.

Another course-grained modeling approach, which from the Hwa group, also considers the size of the proteome as a fundamental limiting factor in heterotrophic microbial growth (Scott et al., 2010) (Figure 2). These models combine the empirical growth rates with an analysis that derives from the long-standing observation that microbial growth rates are linearly proportional to the cellular content of ribosomes (Schaechter et al., 1958). Using this approach, the authors derived a set of quantitative relationships between growth rate, gene expression, and cellular composition in terms of allocation of proteomic resources toward different functional classes of protein (Scott et al., 2010; Scott and Hwa, 2011). The minimal model for heterotrophic bacteria is comprised of a finite proteome consisting of three sectors: a fixed core sector designated “Q” that, for a given species is invariant in proportion of the total proteome, a ribosome sector, “R” of variable size consisting of all ribosomal proteins and their affiliates, and a “P” sector, also variable in size, associated with nutrient uptake. The fixed Q sector might consist of proteins involved and cell maintenance metabolism and the biogenesis of ultrastructure. The sizes of the different sectors were determined experimentally, for example, by manipulation of the growth rate under different nutritional regimes or with different inhibitors. The Q sector was experimentally found to be ~55% of the total proteome for *E. coli* and was not found to change its size across a variety of trophic conditions suggesting that it is, indeed, fixed in proportion of the total proteome. The size of the R sector is variable and directly proportional to the growth rate in accord with the linear relationship between ribosome content and growth rate. Interestingly, the course-grained replicator models of Molenaar mentioned earlier have the linear ribosome-growth rate relation built into them. The third sector, “P” is also variable in size and expands at the expense of the R sector. The P sector exerts a positive effect on growth by providing nutrients that ultimately feed precursors into the R sector. In effect, the P sector “feeds” the R sector with precursors for protein synthesis. However, optimal growth requires a balanced proteomic allocation of the P and R sectors. Increases in the P sector, due, for example, to the necessity of increased nutrient uptake capacity by synthesizing more transporters, occurs at the expense of the R sector and correspondingly results in a lower rate of growth than if less of the proteome were invested in the P sector and more in the R sector. Additional sectors can be elaborated by subdivision of the three sectors of the minimal model and it was shown how the expression of unnecessary protein tends to reduce growth rates due to the reduction in the sizes of the R and P sectors. Extension of this approach has provided new explanatory insight into the role of cyclic AMP in coordinating catabolic and anabolic responses to shifting nutrient regimes (You et al., 2013). In each case, the models necessarily constrain the size or cost of the proteome in order to obtain the far reaching conclusions regarding the way proteomics resource allocation govern maximal growth rates in microbes and how the different functional modules are regulated to achieve this. It is also important to note that each of these cases involves an experimental approach to provide estimates of the sizes of the sectors.

While these approaches have not been applied to cyanobacteria, there is already a rich and advanced set of modeling efforts for algae and cyanobacteria that are oriented toward autotrophic productivity in natural marine and aquatic environments [c.f. Ross and Geider (2009)]. Although, there is likely a huge opportunity to find the parallels between these successful modeling approaches for autotrophs and the FBA and course-grained models discussed above, the main objective of this communication is to discuss the hypothesis that the proteomic constraints that have proven necessary for successful modeling of heterotrophic microbial growth are also of fundamental significance in explaining cyanobacterial growth and productivity.

## Overflow Metabolism is a Dissipative Process That Reflects the Proteomic Constraints of Microbial Growth

It is useful to consider one of the puzzles of microbial physiology: the seeming wastefulness in the phenomenon of metabolic spilling. In heterotrophic microbes, metabolic spilling involves the release of incompletely catabolized energy-rich metabolites and has physiological roles that extend beyond biochemical considerations such as redox balancing. Instead, it appears necessary for maximal growth under certain trophic conditions despite the apparent wastefulness of the process. When heterotrophic cells have an excess of a carbon/energy source they may metabolize this compound using less efficient pathways (e.g., lower ATP yield per substrate) with the consequent wasteful release of energy-rich compounds such as ethanol, acetate, and lactate despite the fact that environmental and physiological conditions would allow for more energy-efficient utilization of the carbon source. For example, the well-known Pasteur effect, which involves metabolic switching between efficient and less efficient pathways. Here, yeast cells are observed to switch between less energy-efficient fermentation and more efficient aerobic metabolism by depending upon the availability of oxygen. However, what is less discussed are the circumstances where the Pasteur mechanism is over-ridden and fermentative metabolism occurs even under aerobic conditions – i.e., overflow metabolism occurs. This situation, known as the Crabtree effect in yeasts (van Dijken et al., 1993), occurs under conditions of excess substrate carbon and is characterized by the operation of fermentation pathways even when oxygen as a terminal electron acceptor is present, potentially allowing the more efficient utilization of substrate. This phenomenon also occurs in bacteria and tumor cells under conditions of heterotrophic substrate excess and, interestingly, under conditions of nitrogen limitation. Although metabolically wasteful, utilization of low efficiency enzymes appears to allow for faster growth under these regimes. Apparently, the wasteful metabolism reflects a tradeoff that also rewards the minimization of costs for the catabolic enzymes, with the cost of the enzymes being either the relative costs of synthesis of the enzymes or the relative costs of cytoplasmic space occupancy by the enzymes of limited cytosolic space (see next section). These costs are summarized by the phrase “proteomic limitation” as modeled in advanced FBA models that take account macromolecular expression (O’Brien et al., 2013) or in course-grained models that explicitly account for the allocation of proteomic resources (Molenaar et al., 2009; Scott et al., 2010; Scott and Hwa, 2011).

Overflow metabolism in autotrophs is less studied than in heterotrophs, but may also play important physiological roles. Though not metabolic energy spilling *per se*, all organisms capable of oxygenic photosynthesis, including cyanobacteria, have the ability to dissipate excess light energy in the form of non-photochemical quenching (NPQ) (Niyogi and Truong, 2013). This involves the dissipation of excitation energy captured by the light-harvesting antennae by regulated mechanisms that convert the excitation energy into heat under conditions where the energy cannot be productively utilized by the photosynthetic reaction centers. Thus, NPQ is a form of energy spilling. Although excitation energy spilling is widely discussed in the area of photosynthesis, the concept of metabolic energy spilling in photosynthetic organisms is less thoroughly considered. In principal, metabolic energy spilling could occur as the release of compounds from the cell or the action of futile cycles that dissipate, for example, excess reductant produced by the light reactions. A physiologically significant example is a futile cycle associated with the CO_2_-concentration mechanism (CCM) of cyanobacteria. The CCM is a metabolic process that not only satisfies the requirement of the inorganic carbon supply by energetically acquiring bicarbonate, but it also dissipates excess energy under high light conditions by spilling of large amounts of acquired bicarbonate by release into the environment (Tchernov et al., 2003). Therefore, in aquatic systems, light may produce a net increase in the amount of bicarbonate dissolved in the media rather than the expected decrease due to uptake by the carbon fixation activity by the Calvin–Basham–Benson (CBB) cycle. Because the CCM is powered by the products of the photosynthetic electron transport (PSET) chain, ATP and NADPH, the excretion of bicarbonate provides an escape valve when the electron transport chain is more active than CO_2_-fixation by the CBB cycle, as under conditions of excessive light. Metabolic spilling in cyanobacteria is also observed in mutants having defects in their ability to synthesize glycogen are observed to excrete pyruvate and α-ketoglutarate into the growth medium, apparently to dissipate excess carbon fixed via the CBB cycle (Carrieri et al., 2012; Gründel et al., 2012). Presumably, this occurs because the assimilatory flux capacity of anabolic metabolism is exceeded by the production of these intermediates and the glycogen synthesis pathway normally absorbs this excess flux of reduced carbon, but in its absence, excretion of pyruvate and α-ketoglutarate occurs.

## “Proteomic Limitation” of Growth Rates is Due to Crowding Limits within the Cell

A common thread that emerges in the different modeling approaches discussed above is the necessity to include a constraint on the size or expression cost of the cellular proteome in order to derive critical features of microbial growth. Only when protein costs are explicitly included in the optimization models do we find that comprehensive predictions of metabolism possible. The case in point is the prediction of overflow metabolism in heterotrophs. Expression of metabolically inefficient, but less costly (protein expression cost) pathways leads to maximal growth rates under conditions of nutrient excess and accounts for the efficacy of overflow metabolism in maximizing cell growth rates. On the other hand, expression of metabolically more efficient (non-overflow) pathways catalyzed by enzymes with a higher proteomic cost, lead to maximal growth rates under conditions of nutrient limitation (Molenaar et al., 2009). However, the expression of more efficient, but more costly enzymes also comes at the expense of dedicating less proteomic resources toward the machinery of protein synthesis (ribosomes and affiliated proteins) so the efficiency of utilizing the limiting nutrient is maximized but only at the simultaneous cost of allocating less of the proteome to ribosomes and cell duplication, which accounts for the lowered rate of growth. This is consistent with the independent line of reasoning and experiment that led to the “bacterial growth laws” showing the allocation of proteomic resources enabled maximal bacterial growth and accounts for diverse regulatory features controlling bacterial metabolism and gene expression (Scott et al., 2010; Scott and Hwa, 2011; You et al., 2013). Similarly, when metabolism and gene expression are simultaneously integrated into the FBA model for *E. coli* growth, overflow metabolism is explained in terms of differing proteomic costs. Furthermore, growth regimes that are distinguished by nutrient limitation at lower growth rates and “proteome-limited” at maximal growth rates, fall out of the analysis (O’Brien et al., 2013). As discussed throughout, this apparent “proteome-limitation” and the associated “proteomic costs,” appear to be a consequence of the physical constraints of intracellular crowding in bacteria. The underlying, yet still unproven assumption, is that expression of metabolically efficient pathways has a higher proteomic cost. Maximal rates of growth occur where all nutrients are in surplus and, under these surplus conditions, intracellular factors place an upper bound on the fastest achievable growth rates. At lower nutrient levels, growth rates are constrained by the limiting nutrient(s) and growth rate follows a dependence on nutrient concentration according to the quasi-Michaelis–Menten formulation of microbial growth (Monod, 1949). Thus, at saturating levels of nutrient (and for autotrophs and saturating light intensities), maximal growth rates are limited by intrinsic factors within the cell, whereas nutrient and light availability limit growth rates below this threshold.

What sets the upper bound on the maximal growth rate rates under saturating nutrient and light conditions for cyanobacteria? There is compelling evidence that the packing density of molecules and the solvent capacity of the cytoplasm along with the packing density of membrane complexes in microbial cell membranes all place severe limits on the size of the proteome in a microbial cell (Beg et al., 2007; Tadmor and Tlusty, 2008; Vazquez et al., 2008; Klumpp et al., 2013). The packing of photosynthetic membranes is very dense in chloroplast (Kirchhoff et al., 2011) and in cyanobacteria (Folea et al., 2008). In other words, the cost of a protein can be evaluated in terms of the space it occupies because expression of that protein comes at the expense of limiting the available cytoplasmic or membrane space allocated for the expression of other proteins. Atkinson argued that one of the fundamental selective constraints in the evolution of cells was the conservation of “solvent capacity” with the view that enzymes are optimized to be efficient, in part, to avoid “wasting solvent capacity” that would otherwise occur by the accumulation of high concentrations of metabolic intermediates (Atkinson, 1977). He argued that natural selection has favored enzymes in a pathway should have lower *K_m_* values to have the fast substrate-product conversion fluxes even when metabolite concentrations are low. With such efficient enzymes, high flux rates through metabolic pathways can be maintained even at low metabolite concentrations. Of course this also assumes that the utilization of products of those pathways is equally efficient to avoid product accumulation. But this gets back to the larger point of proteome allocation: if the product of a biosynthetic pathways composed of efficient enzymes is an amino acid, then it would important that the expression of protein synthetic machinery is maintained at a high enough level (fraction of the proteome) to utilize the amino acids being produced. Conversely, if there is not a large enough allocation of amino acid biosynthetic enzymes (or the enzymes feeding nutrient precursors to those biosynthetic enzymes), then the proteomic allocation to protein synthetic machinery would be excessive, and in a cellular environment where to total proteome is finite, the allocation would be suboptimal. The concentration of soluble protein in the cyanobacterial cytoplasm is estimated to be ~500 mg/mL (Moal and Lagoutte, 2012), similar to the high concentrations estimated for other bacteria (Zimmerman and Trach, 1991; Cayley et al., 2000; Ellis, 2001; Cayley and Record, 2003), although accurate estimates are technically challenging. The dense crowding of the bacterial cytoplasm is mainly due the volume occupied by protein and RNA, with the latter mostly associated with ribosomes (Neidhardt et al., 1990). The proportions of protein, RNA and metabolites represent about 55, 20, and 3% of the dry mass of the cell (Neidhardt et al., 1990). Nevertheless, the impact of macromolecular crowding on the metabolome may be critical because of the limitation it imposes upon the availability of water for solvation of metabolites (Cayley et al., 2000; Cayley and Record, 2003). That said, macromolecular diffusion rates are disproportionately decreased by crowding relative to small smaller solutes (Mika et al., 2010).

Macromolecular processes may be rate-determined by crowding limitations and this may affect the rate that the components of the cell can be duplicated. These limitations can be expressed in terms of characteristic transit times for the occurrence of productive collisions of metabolic and macromolecular reactants, which is controlled by the intercellular diffusional parameters involved. Theoretical analysis using this approach and using the simplifying assumption of spherical cell shapes, calculates the optimal size for a generic bacterial cell to be slightly larger than one micron, close to actual sizes in nature (Soh et al., 2013). For example, the highly crowded nature of the bacterial cytoplasm requires the expression of high concentrations of translation factors such as EF-Tu (Klumpp et al., 2013). This is because molecular crowding restricts the macromolecular diffusion of translational complexes that cooperate with the ribosome in protein synthesis and thus high concentrations are required to overcome this diffusional restriction and to achieve maximal cell growth rates (Klumpp et al., 2013). The attachment and localization of the enzyme ferredoxin NADP reductase (FNR) to the phycobilisome rods (Schluchter and Bryant, 1992) places it in proximity to PSI, which is calculated to be critical for the fast reduction of NADP^+^ by linear electron transport (Moal and Lagoutte, 2012). Again, a mechanistic strategy has evolved to overcome diffusional limitations, this time realized by localizing the reactants in close proximity to one another. Recently, chemical analysis of the plastoquinone pool in cyanobacteria has indicated that the overall balance of the reduced and oxidized forms of plastoquinone are remarkably stable, which is in stark contrast to the dynamic changes evident in the fluorescence transients attributed to the redox state of the plastoquinone pool’s ability to re-oxidize the electron acceptor side of photosystem II following its reduction by photochemical charge separation (Schuurmans et al., 2014). Conceivably, this disparity may reflect the spatial inhomogeneity in the redox state of the plastoquinone population with the reduced form accumulating near photosystem II at a rate faster than the rate that it can diffuse away to the cytochrome b_6_f complex for oxidation. Overall, crowding of the cytoplasm and membrane systems with macromolecules suggests that the necessary modeling constraint of “proteome-limitation” discussed above may have a physical basis in terms of available space in the cytoplasm and membrane. This space limitation hypothesis is in contrast to the other alternative constraint hypothesis expressed in terms of the costs of ATP expenditure required for protein synthesis or limitations in growth due to the energetic costs for macromolecular precursor synthesis (Akashi and Gojobori, 2002). While metabolic efficiency may be at a premium under certain circumstances such as low nutrient environments (Molenaar et al., 2009; O’Brien et al., 2013), it is likely that the highly crowded cellular milieu places a premium on the space and solvent capacity occupied by the myriad molecular constituents of the cell.

While there is a good argument that proteomics costs in the form of limited space constrain maximal growth rates, one can ask whether the same constraints apply to growth in the nutrient-limited region of the growth versus nutrient availability curve? It is possible that under certain conditions, the expression of additional nutrient uptake and assimilation proteins could partially or entirely alleviate the nutrient limitation, but this up-regulated expression may begin to compete for cellular occupancy space with other important functional classes of protein. Nevertheless, there are also conditions where no amount of increased expression of uptake proteins can alleviate a deficiency of an essential nutrient if the nutrient is present only in vanishingly small amounts. Thus, it is conceptually useful to consider the two domains of growth limitation as metabolically limited and proteomically limited (Figure 1), referring to the nutrient-limited submaximal growth rate and the nutrient saturated maximal growth rate, respectively (O’Brien et al., 2013).

## Hypothesis: Proteomic Limitations within the Cyanobacterial Cell Constrain Maximal Growth Rates and Photosynthetic Adaptation

The overall hypothesis is that growth rates are constrained by the limits on the total amount of proteins and other macromolecules that can be fit inside a cyanobacterial cell. Space and crowding constraints combined with the restrictions on surface-to-volume ratios are hypothesized to be the fundamental physical restrictions on the composition and function of the microbial cell, including cyanobacteria. As suggested below, this results in a novel and testable explanation for reductions in growth under conditions of osmotic stress. Surface-to-volume ratios, combined with cell shape, determine the capacity for nutrient and waste exchange across the cell boundaries and give an upper limit on the size of microbial cells (Soh et al., 2013). Packing, solubility, and solute concentrations limit how much material can be confined within the cell volume and membrane domains, which thereby limits the sum total of expressed proteomic resources, as discussed above. In effect, optimizing the allocation of protein resources for maximal cell growth rate is a zero sum game in the sense that production of more proteins in one functional class occurs at the expense of the ability to produce proteins in another functional class. This restriction is explicitly accounted for in the phenomenological models that minimally partition the proteome into three different sectors, the Q, P, and R sectors with the relative proportions determined by the growth rate (Scott et al., 2010; Scott and Hwa, 2011; You et al., 2013). This proteomic restriction is also explicitly incorporated into the course-grained models of heterotrophic microbial growth (Molenaar et al., 2009) and the extended detailed FBA models of *E. coli* growth (O’Brien et al., 2013). Moreover, this leads to a prediction regarding the effects of osmotic stress on growth rates in cyanobacteria and other microbes as suggested recently (Klumpp et al., 2013).

### Hypothesis 1

Lowered rates of cell growth in salt-adapted cultures are due to global reductions in the concentrations of macromolecules and metabolites in the cytoplasm. There is strong evidence that growth rates in *E. coli* are inversely proportional to osmotic stress and that this is due to molecular crowding that is exacerbated by the necessity for the accumulation of compatible osmolytes in the cytoplasm (Cayley et al., 2000; Cayley and Record, 2003). During osmotic adaptation, *E. coli* cells enlarge in volume, keeping the protein content per cell relatively constant. The cells increase potassium and osmolyte and also increase the total amount of water per cell (Zimmerman and Trach, 1991). Combined with the proposal that diffusion of critical macromolecules (e.g., EF-Tu) limits growth rates (Cayley et al., 2000; Cayley and Record, 2003; Klumpp et al., 2013), the dilution of these critical molecules due to greater cell volume may account for the lower growth rates. There is a wide diversity of osmotic and salt tolerance characteristics and adaptive mechanisms among different cyanobacteria (Hagemann, 2011). Although information on changes in cyanobacterial cell size following adaptation to osmotic stress seems to be scant, at least one instance of larger cell size has been reported (Erdmann et al., 1992). Salt-tolerant and halophilic cyanobacteria generally synthesize compatible osmolytes (e.g., glucosylglycerol in *Synechocystis* sp. PCC6803) and accumulate potassium to maintain osmotic balance when challenged by high salt conditions. Immediately following an upshift in environmental salt concentrations, there is a decline in metabolic activities, such as in PSET, and there is a mobilization of many stress response genes. After this initial period, compatible osmolytes accumulate, damage is repaired and cyanobacterial cells regain nearly normal levels of metabolic activities and resume growth, albeit at a lower rate (Hagemann, 2011). The crowding of the cytoplasmic volume due to the accumulations of high concentrations of potassium and compatible osmolyte, while essential for osmotic adaptation, is hypothesized to reduce the growth rate of the cells due to occupancy of solvent space. Analysis of molecular crowding with respect to the concentration of translation factors necessary to sustain high growth rates in bacteria led to a similar hypothesis regarding the effect of increasing external osmotic stress (Klumpp et al., 2013). One of several predictions is that osmotic-stress adapted cyanobacterial cells ought to have either lower amounts of protein per cell, or similar amounts of protein per cell, but cells with larger volumes, as with *E. coli* (Zimmerman and Trach, 1991) and the resultant dilution of cellular protein should correlate with reductions in growth rate.

## Hypothetical Modeling of the Consequences of Proteomic Limitations in the Growth and Allocation of Proteomic Resources in Cyanobacteria

**Figure 3:**
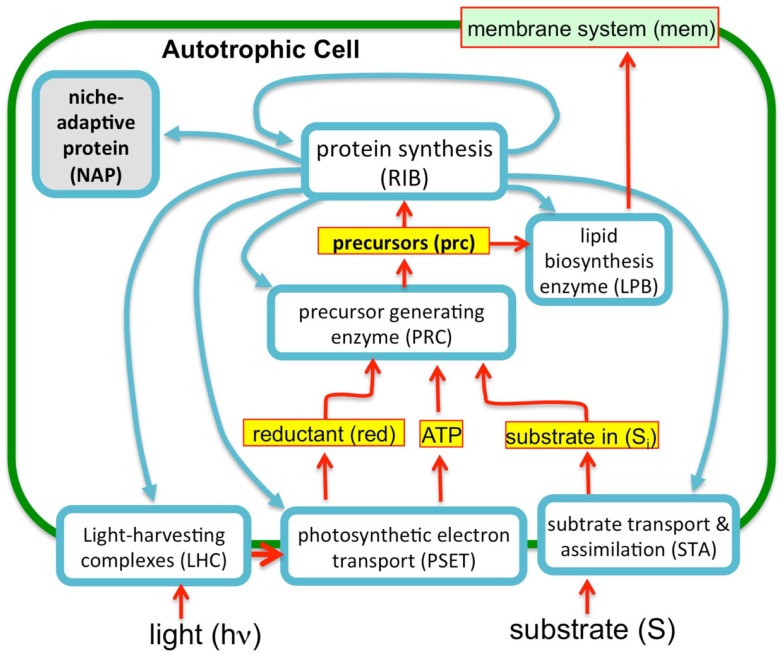
**A simplified autocatalytic replicator model of cyanobacterial growth**. The model consists of a simple set of enzymes (blue outlined boxes), metabolites (yellow boxes), and membrane structural components (green objects) representing functional classes of molecules (e.g., enzyme “ribosome” represents are ribosomal proteins and those affiliated with protein synthesis). The proteins interrelated by a stoichiometric matrix and kinetic equations as described in the text. The model optimizes the allocation of finite proteomic resources among the different proteins (as protein synthesis, blue arrows) to achieve maximal growth rates. Consistent with the observation that microbial growth rates scale in direct proportion to the number of ribosomes, this “ribosome centric” model defines a set of “enzymes” (light blue boxes) that feed precursors to ribosomes and are, in turn, subject to synthesis by the ribosomes. Growth rates correspondingly correlate with flux rates for the generation of precursors for protein synthesis. The model was implemented in GAMS software environment (Andrei, 2013) using a previous model of heterotrophic growth (Molenaar et al., 2009).

To evaluate the imposition of the constraint of a finite proteome in relation to photosynthetic growth of a microbe, an optimization model was constructed based upon the “autocatalytic replicator” models of Molenaar et al. (2009). The original publication provided the source code of the models and this code was re-structured to emulate, in a highly simplified manner, autotrophic metabolism. In contrast to the highly detailed models used in FBA, these models are “course-grained” models. The models are implemented in the GAMS language (Andrei, 2013) and were set up to compute the optimal allocations of proteins necessary to achieve maximal growth rates (Molenaar et al., 2009). The original heterotrophic microbial growth model consists of a simple set of relations between enzymes and metabolites and is arranged in a way that satisfies basic assumptions of balanced cell growth, cell volume, and composition, and are defined by a set of equations and corresponding stoichiometry matrix (Molenaar et al., 2009). The present model uses the same strategy, but involves a rudimentary model for autotrophic metabolism (Figure 3). In essence, the previous heterotrophic model and present autotrophic model each represent a system of enzymes and transporters that feed precursors to ribosomes and membrane lipids. The GAMS language allows definition of mathematical sets and subsets, such as proteins and enzymes, allowing for versatile configurations and operations in the model, which in this case involved optimization of the size of the subsets. A ribosome subset of the proteins generates all proteins and the system is autocatalytic in the sense that ribosomes beget more ribosomes. This is possible due to the cooperation of ribosomes with the other proteins that contribute to the generation of precursor (e.g., amino acids) feeding the ribosomes and membrane structures. In these highly simplified models, the “ribosome enzyme” actually represents all proteins involved in protein synthesis, or in the terminology of the Scott models, the R sector (Figure 2), which is comprised of all “ribosome affiliated” proteins. The system of equations includes expressions that describe the different allocations of active ribosomes toward different sets of enzymes (substrate assimilation, activating enzymes, and the ribosomes themselves). In other words, the total pool of ribosomes sums to one with different fractions of the total ribosome pool working on different subsets of protein. The fractional distribution of ribosomes working on the synthesis of different subsets of proteins is optimized in these models with the objective function being the maximization of growth rate, μ. The actual proportions of the enzymes produced during the optimization of the model are not likely quantitatively accurate since a fitting of real kinetic parameters was not attempted either in the original or present models. However, in the case of the original heterotrophic models, the overall trends were informative since, for example, the switch between non-overflow and overflow metabolism could be emulated and the notion of a proteomic constraint was crucial, as discussed above. In the current form, substrate uptake involves the aggregate of all potentially limiting inorganic substrate transport and assimilation proteins (STA) and a PSET chain that generates ATP and reductant. The goal of the model is, essentially, to feed precursors to the ribosomes. The products of the STA and PSET proteins are combined by the action of precursor biosynthesis enzymes (PRB). The main difference with the original models of Molenaar et al. is the mechanism to generate the pool of precursors (prc) necessary for the synthesis of protein and lipid, which is now satisfied by the parallel action of the PSET and STA proteins. Collectively, these constitute the “core” proteins of the autotrophic replicator. In addition to these core proteins, a subset of proteins called “niche-adaptive proteins (NAP)” were modeled here. These are supposed to represent species-specific proteins that enable the organism to thrive in a particular niche and may not be present in other species. An additional difference with the heterotrophic models is the assumption that, apart from light, the external substrate is not a source of energy. As with the original model, there are constraints in the model to provide limits on the size of the cell, the fraction of proteins occupying the lipid membrane and cytoplasm. The model is schematically shown in Figure 3.

## Effects of Light Intensity on Growth Rates at Different Concentrations of Available Inorganic Substrate

**Figure 4:**
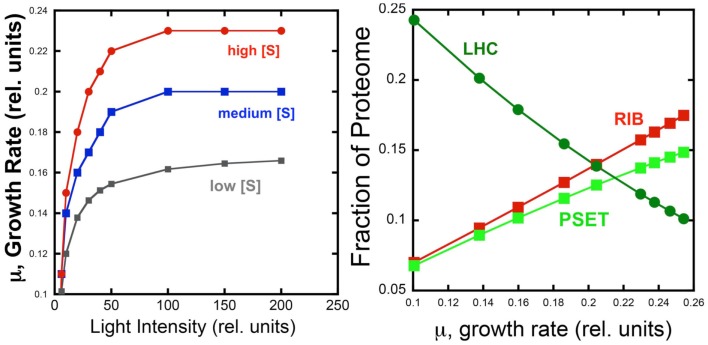
**Simulated effect of light intensity and inorganic substrate concentration on photoautotrophic growth rate and proteome allocation**. Growth rates increase with light intensity, but rates saturate at different levels set by different levels of substrate concentration (Left). Allocation of the proteome to ribosomes (RIB) and photosynthetic electron transport (PSET) and light-harvesting complexes (LHC) as a function of growth rate (Right Panel) using data from the high light simulation shown in the left panel.

The behavior of the model under different light and substrate conditions was explored by iteratively varying these two environmental conditions. Each iteration computed the optimal distribution of proteomic resources among each of the different enzyme groups with the overall mathematical objective of maximizing the growth rate. Figure 4 shows the increase in growth rate as a function light intensity at three different substrate levels. The simulated growth rate as a function of light intensity was observed, as expected, to exhibit a saturation behavior that is modulated by substrate availability. Although the model is too simple to specify it, the substrate could represent inorganic carbon and each of the three curves would represent the light saturation behavior of autotrophic cell as a function of inorganic carbon availability. Besides this lack of specificity, the present model has the additional limitation of not considering important physiological characteristics, such as the photosystem II to photosystem I ratio, which are known to be regulated as a function of irradiance and inorganic nutrient availability. Nevertheless, the current model does provide a first approximation of predicted optimal physiological responses to alterations in environmental conditions. When the allocation of the proteome is examined for one of the curves (high light) in this computational experiment, it is found that the fraction of light-harvesting complexes (LHC) decreases when plotted as a function of growth rate. This is expected since light is limiting growth early in the light saturation curve and at low growth rates with these conditions is reflected by the near linear decrease in the fraction of the proteome composed of LHC due to the fact that a smaller antenna provides the sufficient excitation of the PSET. It is worth noting that PSET is also predicted to be changing in relative abundance as a function of growth rate during light-limited growth, so that the amount of LHC providing excitation energy is adjusted to a “moving target” of different PSET levels. The model predicts that PSET increases with increasing growth rates, which fits with the expectation that it should parallel the rate of carbon fixation and thus biomass accumulation *in vivo*. A similar predicted increase in the allocation of proteome toward PSET is also observed for substrate limitation simulation (not shown). Since most chlorophyll in cyanobacteria is associated with reaction centers, the prediction of higher PSET with increasing growth rates leads to the prediction that faster growing cyanobacteria should have higher chlorophyll contents on a per cell basis. Evidence for higher levels of chlorophyll per cell in cyanobacteria have indeed been observed for light-limited (Deblois et al., 2013) and nitrogen-limited growth of marine cyanobacteria, at least in the range of moderate to high growth rates (Dittrich, 2013). It is also worth noting that while the predicted proteomic allocations of model may eventually be more directly tested experimentally, the model is mute on the regulatory features that lead to optimal distribution of the proteome amongst the functional sectors. Consistent with the growth hypothesis regarding ribosome content in direct proportion to μ (Schaechter et al., 1958; Worden and Binder, 2003), the allocation toward the RIB fraction of the cell increases linearly with growth rate.

### Hypothesis 2

Species-specific differences in the maximal growth rates of cyanobacteria are due to different proteomic allocations into niche-specific proteins.

**Figure 5:**
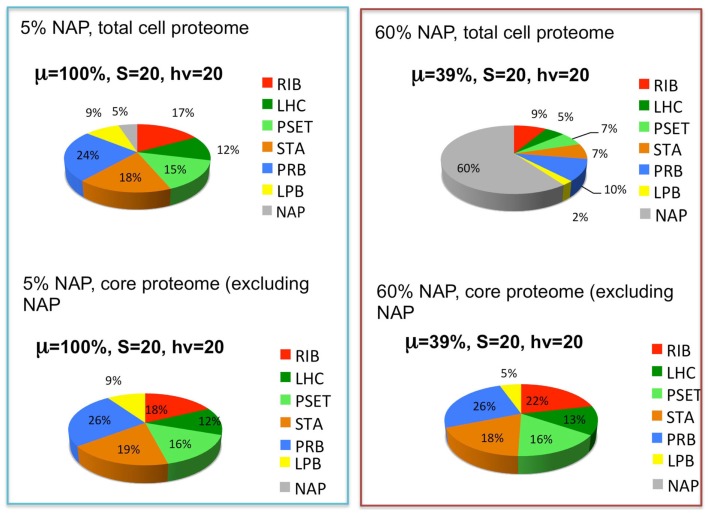
**The effects of the expression of non-core, “niche-adaptive protein (NAP)” on growth and expression of core autotrophic functions**. Upper graphs depict the simulated distribution of the proteome holding the NAPs at 5% (upper left) or 60% (upper right) of the total proteome and the corresponding growth rates (μ) at saturating levels of light (hν) and substrate (S). Lower graphs show that the relative proportions of the other sectors are similar despite the reduction of their net amount due to displacement by the NAPs. Sectors correspond to functional protein groups: inorganic substrate transport and assimilation proteins (STA), photosynthetic electron transport chain (PSET) that generates ATP and reductant, precursor biosynthesis enzymes (PRB). The main difference with the original models of Molenaar et al. (2009) is the energy source (light) and mechanism to generate the pool of precursors (prc) necessary for the synthesis of protein and lipid, which is now satisfied by the parallel action of the PSET and STA proteins. Collectively, these constitute the “core” proteins of the autotrophic replicator (see Figure 3).

The reported doubling times of cyanobacteria ranges from ~3 h to one or more days. We can use the above considerations to formulate the following hypothesis for why different species of cyanobacteria have different maximal growth rates: depending upon the niche they are evolutionarily adapted to, different species may have more or less total allocation of protein resources to NAPs. Hypothetically, cyanobacteria that are adapted to complex environments will need to express additional proteins beyond the core set of proteins needed for autotrophy. Cyanobacteria with smaller “fixed” fractions of NAPs will have the capacity for faster growth because they will be able to devote a greater fraction of the proteome to this core set of proteins, although they would correspondingly have less capacity to adapt to non-ideal environments. The fastest growing cyanobacteria would thus have the most proteomic resources devoted to core functions of autotrophic metabolism and minimal allocation of proteomic resources to specialized nutrient uptake and assimilation, defense mechanisms, and other NAPs. To simulate this, the model (Figure 3) includes a fraction of the proteome that is fixed and representing the NAP. To explore this idea, the simulation was performed at two different levels of NAP with the outcome showing that a large investment in NAP indeed predicts a large decrease in growth rate as might be expected (Figure 5, top panels). This observation is formally identical to the production of heterologous protein in the original heterotrophic models (Molenaar et al., 2009). A further observation is that the relative proportions of the other proteomic sectors is fairly similar under in the high NAP and low NAP cell types if the allocation to NAP protein is excluded (i.e., if the RIB, LHC, PSET, STA, PRB, and LPB are summed to 100%) (Figure 5, bottom panels). This suggests that the metabolic balance between these functional sectors is balanced in each cell type and that all sectors “expand” when the NAP proteins are removed from the optimization. Although not modeled, deviations from this trend would be anticipated to the extent that the NAP proteins utilize additional cellular resources (e.g., reductant) beyond the synthesis of the precursors that go into forming the NAP class of proteins.

**Figure 6:**
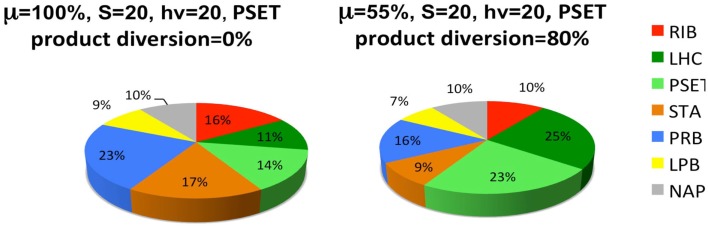
**Simulated diversion of energy precursors to excreted products alters the allocation of the proteome**. Product diversion is defined as the excretion of 80% of the energy precursor for engineered product synthesis. Only ATP and reductant are considered in this highly simplified model, whereas a more realistic model will depend upon the chemical characteristics of the excreted product, most notably, the C/H ratio of its chemical formula. See Figure 5 and text for definitions of the functional protein groups of the model.

The last set of simulations that were performed to investigate the consequence of engineering cells to divert metabolic precursors toward an excreted “energy” product (Figure 6). This scenario might apply to cells that are engineered for biofuel production, for example. The simulation is highly simplified in the sense that it only considers the diversion of ATP and reductant toward a hypothetical excreted product and ignores the more realistic inclusion of diversion of a fraction of the material substrates (S) toward this end. A more realistic model will result in proteome allocations that depend upon the chemical characteristics of the excreted product, most notably, the C/H ratio of its chemical formula. Nevertheless, the findings are interesting and show that under these simplified circumstances, the model predicts a resultant re-distribution of the proteome in accord with what is observed in experiment. It has been shown that the cyanobacteria engineered to excrete sugar have adjusted their metabolism to have increased total photosynthetic capacity presumable to compensate for the genetically imposed drain on their metabolism (Ducat et al., 2012).

## Conclusion

The statistician, George Box stated that “essentially, all models are wrong, but some are useful” (Box and Draper, 1987). The autotrophic replicator model (ARM) is probably best considered a preliminary construction should be useful for bringing current ideas on heterotrophic microbial growth to the topic of autotrophic growth. It should also be useful since testable hypotheses can be derived from the model and packing constraint assumption presumed by the model. The present model was developed on the basis of previous models where it was concluded that inclusion of “macromolecular costs” is critical for accurate representation of optimal cellular metabolism and this is evident when trying to account for heterotrophic overflow metabolism (Molenaar et al., 2009). This constraint was also included in the ARM. It was also concluded that the best hypothesis for macromolecular costs was probably not the energy costs, but rather packing constraints limiting the size of the proteome. Accordingly, allocation of proteomic resources is essentially a zero sum game with the consequence that increased investment in proteins for niche adaptation, for example, result in slower growth rates due to correspondingly smaller investments in proteomic resources dedicated to cell duplication (e.g., ribosomes and energy generation). Proteomic limitation of maximal growth hypothesis lends itself to experimental tests including growth slowdowns due to loss of solvent space during osmotic stress as surmised, but not tested earlier (Klumpp et al., 2013). Such thinking also provides a heuristic explanation to better explain why some strains of cyanobacteria and algae exhibit much faster maximal growth rates than others. The autotrophic lifestyle requires an enormous investment in the synthesis of photosynthetic membranes and the enzymes of inorganic carbon uptake and carbon fixation. The ARM formulated is useful for glimpsing of trends in adaptation and engineering in cyanobacteria that can later be more accurately obtained from more sophisticated FBA models or from refinement of the current models to more accurately represent the kinetic and stoichiometric relationships anticipated in a real cyanobacterial cell. Besides the growth hypothesis regarding the linear relationship between ribosome number and growth rate, additional testable predictions can be made including the abundance of photosynthetic machinery as a function of growth rate, species type, and environmental condition. Improvements would also include modeling different ATP and NADPH demands under different conditions and the corresponding ability to adjust the (photosystem I/photosystem II) ratios. It would also need to include alternative electron transport (e.g., cyclic electron flow) and specific nutrient uptake mechanisms such as the CO_2_ concentrating mechanism. Such considerations are already made in FBA approaches (Knoop et al., 2010; Nogales et al., 2012), which would then need to be extended to include macromolecular expression (FBA-ME) (O’Brien et al., 2013). Furthermore, improvements can be made by trying to utilize the rich modeling literature regarding cyanobacterial and algal growth that have been effectively applied especially for understanding growth dynamics in marine environments (Ross and Geider, 2009).

Optimal growth ultimately requires that allocation of the proteins constituting different functional modules of the proteome result in a set of flux balances between the production and utilization of cellular metabolites. It has been argued here that the main reason for protein allocation being a zero sum game is that molecular crowding places a ceiling on the amount of protein in a bacterial cell. In the ARM, the amount and activity of the PSET proteins is assumed to be sufficient to supply ATP and reductant at rates that match the demand of enzymes involved in the generation precursors (PRC) for macromolecular synthesis. Natural selection has tuned the regulation to ensure optimal protein levels to achieve this balance. As noted, the ARM does not specify mechanisms, but it seems likely that metabolic intermediates that accumulate or are depleted under conditions of imbalance are good candidates to serve as allosteric modulators of gene expression. This type of regulation is observed, as one example, for control of the inorganic carbon uptake mechanism proteins (Nishimura et al., 2008; Daley et al., 2012). It is also almost certain that other selective forces modify expression levels of proteins to not only satisfy flux balance, but also achieve system robustness. For example, the PSET appears to be expressed at levels that exceed the amounts needed to supply its main products, ATP and NADPH, to the CBB cycle based upon measurement of photochemical quenching capacity at steady state ambient growth light conditions. One possibility is that expression levels of the PSET proteins have evolved to cope with fluctuations in light intensity and have resulted higher expression levels to be able to the PSET to absorb peaks in energy input without the generation of damaging reactive intermediates.

## Conflict of Interest Statement

The author declares that the research was conducted in the absence of any commercial or financial relationships that could be construed as a potential conflict of interest.

## Supplementary Material

The Supplementary Material for this article can be found online at http://www.frontiersin.org/Journal/10.3389/fbioe.2015.00001/abstract

## Footnotes

^1^As far as the author is aware, information on possible changes in the total size of the metabolome as a function of different conditions is not readily available.

^2^However, this situation has dramatically changed with the development of FBA metabolic models that have been extended to include gene expression and proteomic constraints, which correspondingly gives predictions for conditions where overflow metabolism occurs (O’Brien et al., 2013).
