# Supplementary material for: Systems and Photosystems: Cellular Limits of Autotrophic Productivity in Cyanobacteria
Source: Front Bioeng Biotechnol. 2015 Jan 20;3:1. doi: 10.3389/fbioe.2015.00001 (PMC4299538; doi:10.3389/fbioe.2015.00001)
Supplement: Supplementary file 1 [file Data_Sheet_1.DOC]

$ontext

This is a reconfiguration of the orignal models of a hetertrophic cell growth published in the article:

Molenaar, D., Van Berlo, R., De Ridder, D., and Teusink, B. (2009).

Shifts in growth strategies reflect tradeoffs in cellular economics. Mol Syst Biol

To learn more on how to run the model and obtain the GAMS software, please see the supplemental material that is associated with that article.

http://www.nature.com/msb/journal/v5/n1/suppinfo/msb200982_S1.html

The present model simulates an autotrophic cell.

The cellular reaction scheme is as follows:

RIB

------> proteins

LHC PSET PRB /

hv -----> hvi --------> red ------->p

/ \ / / \ \-------> lipids

ADP ATP ATP / ADP LPB

/

S -----> Si

STA

$offtext

SETS

cmp "all cell components" / LHC "light harvesting complex"

RIB "ribosome"

PSET "photosynthesis electron transport enzyme"

STA "substrate transport and assimilation enzyme"

PRB "precursor generating enzyme"

LPB "lipid biosynthesis enzyme"

hvi "absorbed light (hv)"

red "photosynthetic reductant"

Si "assimilated intracellular S"

prc "precursor for protein and lipid biosynthesis"

lip "lipid"

NAP "niche adaptive protein" /

pro(cmp) "all proteins" / LHC, PRB, PSET, STA, RIB, LPB, NAP /

enz(pro) "enzymes" / LHC, PRB, PSET, STA, RIB, LPB /

ATPc(enz) "ATP consuming enzymes" / STA, PRB /

ADPc(enz) "ADP consuming enzymes" / PSET /

memP(pro) "membrane located proteins" / STA, LHC /

mem(cmp) "all membrane located components" / LHC, STA, lip /

met(cmp) "metabolites" / hvi, Si, red, prc, lip /

intM(cmp) "intracellular metabolites" / hvi, red, prc, Si /

intP(pro) "intracellular proteins";

intP(pro) = not(memP(pro));

Display intP;

PARAMETERS

kcat(enz) "kcat of enzyme"

/LHC 7

PRB 5

RIB 3

PSET 10

STA 5

LPB 5/

Km(enz) "Km of enzyme"

/LHC 10

PRB 1

RIB 1

PSET 1

STA 10

LPB 5/

KmATP(ATPc) "Affinity constants for ATP"

/PRB 1/

KmADP(ADPc) "Affinity constants for ADP"

/PSET 0.5/

KmSi "affinity constant of PRB for Si" /PRB 1/

Pmax "maximal intracellular protein concentration" /1/

PLmax "maximal membrane protein/lipid ratio" /1/

Mmax "maximal total metabolite concentration" /2/

sA(mem) "specific surface of membrane located component"

/LHC 1

lip 1

STA 1/

cAXP "total concentration of energy intermediate" /1/

gamm "energetic efficiency of the catalytically efficient catabolic enzyme" /1.0/

hv "extracellular S concentration"

S "external substrate concentration"

prccost "precursor cost in terms of ATP" /1/

* Npr MUST be between 0 and 1

Npr "fraction to niche adaptive protein production" /0.1/;

PARAMETER

table stoich(met, pro) "reaction stoichiometry matrix"

LHC PRB RIB PSET STA LPB

Si -1 1

hvi 1 -1

prc 2 -1 -1

red -1 1

lip 1;

POSITIVE VARIABLES

a(pro) "fraction of ribosomes engaged in synthesis of protein X"

beta "volume to surface ratio"

c(cmp) "concentration of component"

v(enz) "catalytic rate of enzyme"

cATP "concentration of charged energy intermediate"

cADP "concentration of uncharged energy intermediate";

FREE VARIABLE

* Since the objective variable has to be a "free" variable i.e. defined

* on the <-INF,+INF> interval, we transform mu to the logarithmic scale

* So, exp(logmu) = mu and maximizing logmu is equivalent to maximizing mu.

logmu "natural logarithm of the specific growth rate";

EQUATIONS

volume "intracellular volume is determined by beta and the membrane surface"

alphaSum "fractions of ribosomes engaged in synthesis of all proteins sum up to 1"

Pbal(pro) "balance for proteins"

Mbal(met) "balance for metabolites"

balATP "balance for ATP"

balADP "balance for ADP"

cat_RIB "catalytic rate of ribosome pool"

cat_LHC "catalytic rate of hv transporter pool"

cat_PRB "catalytic rate of precursor generating enzyme pool"

cat_PSET "catalytic rate of metabolic enzyme pool"

cat_STA "catalytic rate of metabolic enzyme pool"

cat_LPB "catalytic rate of lipid biosynthesis enzyme pool"

maxP "maximal intracellular protein concentration"

*maxM "maximal total metabolite concentration"

membr "membrane integrity condition"

NAPpr "niche adaptive protein production";

volume.. beta*Sum(mem,sA(mem)*c(mem)) =E= 1;

alphaSum.. Sum(pro,a(pro)) =E= 1;

Pbal(pro).. a(pro)*v('RIB') - exp(logmu)*c(pro) =E= 0;

Mbal(met).. Sum(enz, stoich(met,enz)*v(enz)) - exp(logmu)*c(met) =E= 0;

balATP.. gamm*v('PSET') - prccost*v('PRB') - exp(logmu)*cATP =E= 0;

balADP.. prccost*v('PRB') - gamm*v('PSET') - exp(logmu)*cADP + exp(logmu)*cAXP =E= 0;

cat_RIB.. v('RIB') =E= kcat('RIB')*c('RIB')*c('prc')/(Km('RIB') + c('prc'));

cat_LHC.. v('LHC') =E= kcat('LHC')*c('LHC')*hv/(Km('LHC') + hv);

cat_PRB.. v('PRB') =E= kcat('PRB')*c('PRB')*c('red')*c('Si')*cATP/(c('red')*c('Si')*cATP+ Km('PRB')*cATP + KmATP('PRB')*c('red')+ KmATP('PRB')*c('Si')+KmSi('PRB')*c('red'));

cat_PSET.. v('PSET') =E= kcat('PSET')*c('PSET')*c('hvi')*cADP/(c('hvi')*cADP + c('hvi')*KmADP('PSET') + cADP*Km('PSET'));

cat_STA.. v('STA') =E= kcat('STA')*c('STA')*S/(Km('STA') + S);

cat_LPB.. v('LPB') =E= kcat('LPB')*c('LPB')*c('prc')/(Km('LPB') + c('prc'));

maxP.. Sum(intP,c(intP)) =L= Pmax;

*maxM.. Sum(intM,c(intM)) =L= Mmax;

membr.. Sum(memP, c(memP)) =L= c('lip')*PLmax;

NAPpr.. a('NAP') =E= Npr;

MODEL CELL /ALL/;

CELL.optfile =1;

*hv = 100;

*SOLVE CELL USING NLP MAXIMIZING logmu;

*$ontext

PARAMETER report(*,*,*) "process level report" ;

SET i "iteration driver" / 1*55 /;

hv=50;

S=2;

* Initial values for ATP and ADP (does not affect result)

cATP.l=cAXP/2;

cADP.l=cAXP/2;

LOOP (i,

SOLVE CELL USING NLP MAXIMIZING logmu;

report('','substrate',i) = S;

report('','hv',i) = hv;

report('','mu',i) = exp(logmu.l);

report('','beta',i) = beta.l;

report('alpha',pro,i) = a.l(pro);

report('conc',cmp,i) = c.l(cmp);

report('rate',enz,i) = v.l(enz);

report('','cATP',i) = cATP.l;

report('','cADP',i) = cADP.l;

hv = hv/1.2;

);

OPTION decimals = 4;

DISPLAY report;

* The command below dumps the results in an excel file

* Uncomment it if you want this to happen

$libinclude xldump report Photosynthetic_Cell2.xls modelPS!a1

*$offtext
